# Ethnobotanical study on medicinal plants in Melit area (North Darfur), Western Sudan

**DOI:** 10.1186/s13002-023-00646-9

**Published:** 2024-01-03

**Authors:** Mohammed Almustafa Yosif Mohammed Muhakr, Ikram Madani Ahmed, Gihan Omer Mohamed El hassan, Sakina Yagi

**Affiliations:** Department of Botany, Faculty of Science, University of Khartoum, P.O. Box 11115, Khartoum, Sudan

**Keywords:** Medicinal plants, Traditional knowledge, Melit area, Sudan

## Abstract

**Background:**

The documentation of ethnobotanical knowledge in Sudan is restricted to specific regions, and there is a far-reaching lack of written information on the traditional use of medicinal plants in other places like Darfur State, in western Sudan. The present study was designed to document the medicinal plants used in traditional medicine of Melit area in North Darfur State.

**Method:**

Ethnomedicinal information was collected from 135 local informants through semi-structured questionnaires. Data were analysed for use value (UV), informant consensus factor (ICF) and fidelity level.

**Results:**

A total of 59 medicinal plants, belonging to 32 families and 55 genera, were recorded for their traditional uses in Melit area. Fabaceae were represented by highest number of species (13) followed by Asteraceae and Malvaceae (4 each) and Poaceae (3). Herbs comprise the main sources (50.8%) of traditional remedies. Fruits and stem bark (17.9% each) were the major plant parts used. Decoction (36.5%) is the most mode of preparation used. *Geigeria alata* was most commonly used species with UV of 2.37. The highest ICF values were recorded for swellings (ICF = 1.00) and respiratory system (ICF = 0.95) categories. Ten plants, namely *Carica papaya, Corchorus trilocularis, Eragrostis cilianensis, Heliotropium sudanicum, Mollugo cerviana, Psiadia punctulate, Rhynchosia minima, Solanum coagulans, Solanum forskalii* and *Tephrosia purpurea,* were cited for the first time as medicinal plants used in Sudan traditional medicine. Resins of *Boswellia papyrifera*, seeds of *Nigella sativa,* pods of *Vachellia nilotica* (syn. *Acacia nilotica*) and clove of *Syzygium aromticum* were used to make different preparations for the treatment of the corona virus.

**Conclusion:**

This is the first ethnobotanical survey conducted in this region which is always suffering from security issues, and results indicated that Melit area harbours high diversity of plants used traditionally to cure different health conditions. The present study aids in conserving such rich heritage, and it is recommended that the newly reported species worth further studying over their phytochemical and biological properties.

## Background

Medicinal plants provide beneficial therapeutic effect in traditional health systems for indigenous communities in the world and serve as an important source of lead molecules for drug discovery. The close interaction between man and nature has led to the accumulation of a wealth of traditional knowledge of medicinal plants’ uses presently recognized as relevant to preserving plant biodiversity and understanding the dynamic relationships between wild plants, social and cultural systems [1, 2]. This traditional knowledge is declining and under risk of disappearance due to the fact that it passes orally between generations besides the disinterest, modernization and change of life style among new generations [3]. The lack of systematic documentation may also contribute to the loss of medicinal plant knowledge, particularly for neglected or non-preferred plants [4]. Thus, the documentation of this knowledge through ethnobotanical surveys is important to preserve this valuable knowledge and valorize priority medicinal plants of high therapeutic potential towards new drug discovery.

Sudan harbours a wealth of plants due to its wide variation in its topography, climate, soil and hydrology with about 3969 species belonging to 135 family and 8430 genera are documented [5]. Due to the present war in the Sudan, the country is facing a great shortage in medical healthcare and essential medicine. This situation besides other factors associated with economic crises as well as traditional faith of communities in traditional medicine put medicinal plants at the core of primary healthcare for humans and their livestock. In fact, home remedies are available in virtually every Sudanese home including those of cities where access to modern medical care is available.

The documentation of ethnobotanical knowledge in Sudan is restricted to specific regions, and there is a far-reaching lack of written information on the traditional use of medicinal plants in other places like Darfur State, in western Sudan. Hegazy et al. [6] reported the plants used in Jebel Marra area, situated in the western part of the middle of Darfur State. Fifty-eight plants were recorded to have multiple uses as food, forage, firewood and from them 53 were used medicinally to cure 18 ailments. However, they did not give detailed information about parts used, ailments treated by each plant and their mode of preparation and application. In fact, due to security issues no recent study concerning the flora of Darfur State was performed, the last study dated at 1990 by Elamin [7]. This part of the country suffered from long war beside many famine crises, and people there rely mainly on traditional medicine to treat different ailments. So, the documentation of the plants used in traditional medicines in this region of Sudan is warranted. Moreover, it is highly likely that many potential medicinal plants could be identified and explored for their potential biological activity. Therefore, the current study was aimed to document the traditional plant knowledge on medicinal uses of plants to cure ailments in Melit area (North Darfur State) in Western Sudan.

## Methods

### The study area

**Fig. 1:**
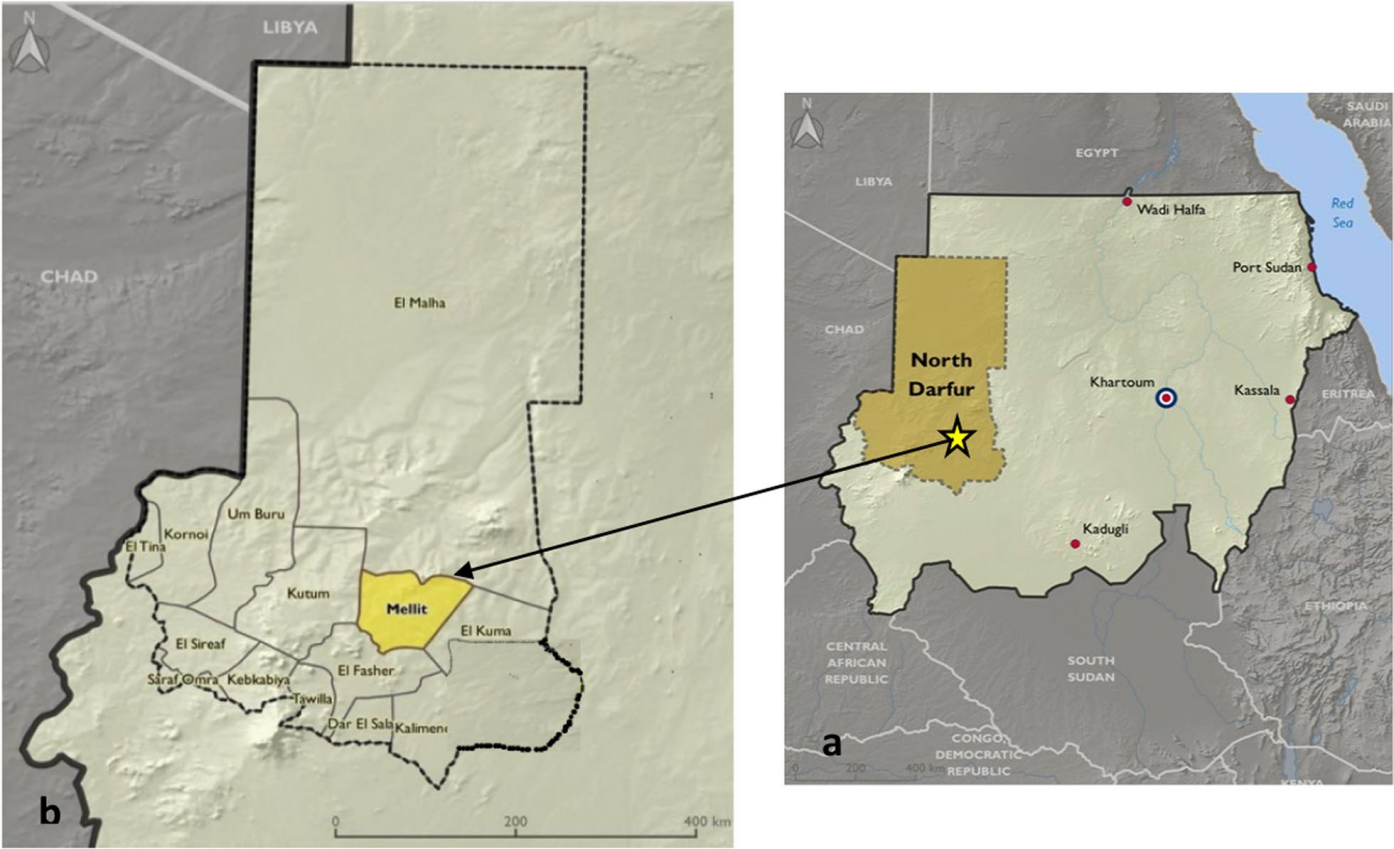
**a** Sudan map showing North Darfur State (brown) and **b** Melit locality (yellow) [8]

The study was carried out in Melit area, North Darfur State in Western Sudan. Geographically, it is located between latitude 14° 08–12° 22N and longitude 25° 32–58° 53 E, with an area estimated at 12.0000 square kilometre (Fig. 1). The region has a semi-arid climate with dry summer and cold winter seasons. In summer, the average of high temperature is 35.5 °C and the minimum average degree in winter is 22.5 °C. The rainy season starts on July–October where the average rainfall ranges from 150 to 350 mm. The general soil classification in Melit locality is 70% sand, 20% gravel and rocky soils, and 10% clay soils. In general, the area is characterized by a flat, sandy plain interrupted by hills and dry wadi beds (dry riverbed that contains water during rainy seasons). The wadi beds are often covered by loamy sands and alluvial soils and in autumn season pour into a giant reservoir tank known as Mellit Khazan. The vegetation cover is made up of scattered shrubs and trees, and during autumn the land is covered by diverse grass species. Melit locality covers five villages, namely Armal, Om Homairon, Armal East, Bamba Tefi and Arid. The total population in Millet, according to the latest Sudan population census in 2009, was 135,831 of which 80% lives in Armal village. The low population density and small size of other villages could be attributed to the hilly nature of the terrain and the poor natural resource base that inhibits population concentration and the growth of large rural settlements. The majority of the population belongs to the Berti ethnic followed by the Baza which constitutes the second largest ethnic group. Other groups present in minority included the Zyadia, Tunjur, Tama, Bargo, Bani Omran, ALbarti, ALzagahaw, Almadoob, ALfoure and Ireigat. They are Muslims and speak Arabic beside their slang languages. The population are fully sedentary, depending on traditional crop farming and animal husbandry. The major crop is millet which serve as the staple food. Other crops like watermelon, hibiscus and cowpeas are usually grown as cash crops. Livestock includes camels, cows, donkeys, goats and sheep. Women account for approximately 60% of the total agricultural labour force, and this number increased dramatically after the eruption of the conflict in 2003 [8].

### Data collection and plant identification

Ethnobotanical data were collected from November 2021 to July 2022 based on semi-structured interviews. A total of 135 informants between the ages of 18 and 85 were interviewed independently to avoid others influence. The questionnaire was designed to collect data on (1) local names of the plants, (2) ailments treated by the plant, (3) plant parts used, (4) condition of the plant material (dried or fresh) and (5) modes of preparation and administration. Some social factors like the name, age and education level of the interviewed person were also recorded.

### Collection and identification of the plants

Fresh plant specimens were collected using the normal plant collection procedure. Plants were identified by using keys of written floras such as Elamin [7] and Andrews [9–11]. Plants’ names were updated according to www.worldfloraonline.org. Voucher specimens were deposited at the Herbarium of Department of Botany, University of Khartoum.

### Quantitative ethnobotanical data analysis

Data were subjected to ethnobotanical analysis tools including;

#### Use categories

The medicinal plant uses were classified into categories following the standard developed by Cook [12]. Each time a plant was mentioned as “used” was considered as one “use-report.” If one informant used a plant to treat more than one disease in the same category, it was considered as a single use-report.

#### Use value

The relative importance of species known locally was calculated employing the use value (UV) as formulated by Phillips et al. [13]:

$${\text{UV}} = \sum {U_{i} /n}$$

where *U*_*i*_ is the number of use-reports cited by each informant for a given species and n refers to the total number of informants. Use values are high when there are many use-reports for a plant, implying that the plant is important, and approach zero (0) when there are few reports related to its use.

### Informant consensus factor

To test homogeneity of knowledge, the informant consensus factor (ICF) was calculated [14]:

$${\text{ICF}} = N_{{{\text{ur}}}} - N_{{\text{t}}} /\left( {N_{{{\text{ur}}}} - 1} \right)$$

where *N*_ur_ refers to the number of use-reports for a particular use category and *N*_t_ refers to the number of taxa used for a particular use category by all informants. ICF values are low (near 0) if plants are chosen randomly or if there is no exchange of information about their use among informants and approach one (1) when there is a well-defined selection criterion in the community and/or if information is exchanged between informants [15].

### Fidelity level

Because many plant species may be used in the same use category, it is interesting to determine the most preferred species used in treatment of particular ailment, which can be done with the fidelity level (FL) of Friedman et al. [16]:

$${\text{FL}}(\% ) = {\text{Np}}/N \times 100$$

where Np is the number of use-reports cited for a given species for a particular ailment and *N* is the total number of use-reports cited for any given species. High FLs (near 100%) are obtained for plants for which almost all use-reports refer to the same way of using it, whereas low FLs are obtained for plants that are used for many different purposes.

## Results

### Demographic features of informants and source of knowledge

A total of 135 informants were interviewed regarding the use of medicinal plants to treat different ailments (Table 1). Out of this number, 31 were traditional healers. Informants constituted six age groups between 18 and 94 years with the majority (42.96%) being within 60–79 years old and second highest (24.44%) were between 40–59 years old. Men represented 55.56% of informants and women 44.44%. Informants were from different educational backgrounds with the majority (54.81%) were illiterate. Oral transmission from one generation to other (89.63%) was the main way through which informants acquired their knowledge on curing diseases by medicinal plants.

**Table 1:** Demographic data on informants and source of knowledge

Demographic feature	Number (%)
*Gender*	
Women	60 (44.44%)
Men	75 (55.56%)
*Age group*	
< 20 years of age	4 (2.96%)
20–40 years of age	26 (19.26%)
40–59 years of age	33 (24.44%)
60–79 years of age	58 (42.96%)
> 80 years of age	14 (10.37%)
*Educational level*	
University	1 (0.74%)
Secondary	15 (11.11%)
Intermediate	14 (10.37%)
Primary	31 (22.96%)
No schooling	74 (54.81%)
*Source of knowledge*	
Ancestral	121 (89.63%)
Self training	14 (10.37%)

### The plants and their medicinal application

A total of 59 medicinal plants, belonging to 32 families and 55 genera, were recorded for their traditional uses in Melit area (Table 2). Fabaceae (Leguminosae) were represented by highest number of species (13) followed by Asteraceae and Malvaceae (4 each) and Poaceae (3). Apocynaceae, Boraginaceae, Capparaceae, Cucurbitaceae, Myrtaceae, Solanaceae and Zygophyllaceae were presented by 2 species each, while Acanthaceae, Alliaceae, Apiaceae, Arecaceae, Aristolochiaceae, Asphodelaceae, Brassicaceae, Burseraceae, Caricaceae, Combretaceae, Lamiaceae, Lythraceae, Meliaceae, Molluginaceae, Moraceae, Olacaceae, Orobanchaceae, Pedaliaceae, Ranunculaceae, Rhamnaceae and Rubiaceae by one species each.

**Table 2:** Ethnobotanical plants used in Melit area (North Darfur), Western Sudan

No.	Family/plant name (voucher number)	Local name	Habit	Part used	Ailment treated	Mode of preparation and application	UV
	**Acanthaceae**						
1	*Blepharis linariifolia* Pers. (BH/BL1122)	Alsieha	Herb	Whole plant	Urine retention	Decoction, potions	1.00
	**Alliaceae**						
2	*Allium sativum* L (BH/AS1122)	Thoom	Herb	Bulb	Haemorrhoids	Infusion, wash	0.57
	**Apiaceae**						
3	*Foeniculum vulgare* Mill. (BH/FV1221)	Shamar	Herb	Fruit	Stomach ache	Infusion, potions	0.73
	**Apocynaceae**						
4	*Calotropis procera* (Aiton) Dryand. (HB/CP0722)	Usher	Shrub	Bark	Wounds	Powder, sprinkle	0.44
					Haemorrhoids	Poultice	
5	*Leptadenia arborea* (Forssk.) Schweinf*.* (HB/LA0222)	Marrkh	Shrub	Bark	Kidney Stones	Decoction, potions	0.70
	**Arecaceae**						
6	*Hyphaene thebaica* Mart. (HB/HT1121)	Nabag	Tree	Fruit	Hypertension	Infusion, potions	0.81
	**Aristolochiaceae**						
7	*Aristolochia bracteolata* Lam. (HB/AB1121)	Um galagil	Herb	Whole plant	Malaria	Infusion, potions	0.99
					Tooth ache	Poultice, filling tooth cavity	
					Scorpion sting	Freshly crush, rub	
	**Asphodelaceae**						
8	*Aloe sinkatana* Reynolds (HB/AS0322)	Sabbar	Herb	Root	Tonsillitis	Infusion, mouth wash	0.42
	**Asteraceae**						
9	*Ambrosia maritima* L*.* (HB/AM1121)	Damesisa	Herb	Seed	Diarrhoea	Infusion, potions	0.33
10	*Geigeria alata* Benth. & Hook.f. ex Oliv*.* (HB/GA1221)	Algassgas	Herb	Whole plant	Diabetes	Infusion, potions	2.37
					Catarrh	Steam, inhalation	
11	*Psiadia punctulata* Vatke (HB/PP0322)	Tibag	Shrub	Root	Swellings	Poultice	0.50
12	*Pulicaria crispa* (Forssk.) Oliv. (HB/PC1121)	Alrihan	Herb	Whole plant	Stomach pain	Decoction, potions	0.63
	**Boraginaceae**						
13	*Cordia sinensis* Lam. (HB/CS0622)	Andram	Tree	Bark	Wounds	Powder, sprinkle	0.24
14	*Heliotropium sudanicum* F.W.Andrews (HB/HS1121)	Gash alagrrab	Herb	Leaf	Scorpion bite	Freshly crush, rub	0.63
	**Brassicaceae**						
15	*Raphanus sativus* f. aka-daikon (HB/RS1221)	Figile	Herb	Root	Kidney Stones	Decoction, potions	0.32
	**Burseraceae**						
16	*Boswellia papyrifera* (Hochst*.*HB/BP0422)	Tarag tarag	Tree	Gum	Corona virus	Decoction, potions	1.15
					Cold/cough	Decoction, potions	
					Diabetes	Decoction, potions	
	**Capparaceae**						
17	*Cadaba glandulosa* Forssk. (HB/CG0622)	Kourmot	Tree	Leaf	Kalaazar	Poultice	0.66
					Wounds	Poultice	
18	*Maerua crassifolia* Forssk (HB/MC0422)	Sarrh	Tree	Bark	Wounds	Poultice	0.44
	**Caricaceae**						
19	*Carica papaya* L. (HB/CP0122)	Papya	Tree	Fruit	Eczema	Poultice	0.27
	**Combretaceae**						
20	*Terminalia brownii* Fresen*.* (HB/TB0422)	Sobag	Tree	Bark	Rheumatic pain	Smoke fumigant	0.37
	**Cucurbitaceae**						
21	*Citrullus colocynthis (L.)* Schrad.(HB/CC0522)	Hanzal	Herb	Leaf	Rheumatic pain	Poultice	0.73
				Seed	Malaria	Decoction, potions	
					Gonorrhoea	Decoction, potions	
					Scorpion sting	Poultice	
*22*	*Momordica dioica* Roxb. ex Willd. (HB/MD0222)	Al erieri	Herb/creeper	Root	Abortive	Decoction, potions	0.47
	**Fabaceae**						
*23*	*Albizia anthelmintica* (A.Rich.) Brongn*.* (HB/AN1121)	Um Takarny	Tree	Bark	Anthelmintic	Decoction, potions	0.61
*24*	*Bauhinia rufescens* Lam. (HB/BR1121)	Kolkol	Tree	Bark	Diabetes	Decoction, potions	0.30
*25*	*Cassia arereh* Delile (HB/CA1121)	Gaga	Tree	Bark	Malaria	Decoction, potions	1.00
					Evil eye	Smoke fumigant	
*26*	*Rhynchosia minima* (L.) DC. (HB/RM0122)	*Shgr Dabib*	Herb	Root	Snake bite	Freshly crush, rub	0.44
					Rabies	Poultice	
27	*Senegalia mellifera* (Benth.) Seigler & Ebinger (HB/SM0522)	Kitir	Shrub	Bark	Syphilis	Ash, poultice	0.59
28	*Senegalia senegal* (L.) Britton (HB/SS0522)	Hashab	Tree	Gum	Kidney disorder	Decoction, potions	0.96
29	*Senna alexandrina* Mill. (HB/SA1121)	Sanamaka algezo	Herb	Whole plant	Constipation	Decoction, potions	1.17
30	*Senna italica* Mill. (HB/SI1121)	Sana sana	Herb	Whole plant	Constipation	Decoction, potions	0.30
31	*Senna obtusifolia* (L.) H.S.Irwin & Barneby (HB/SO0522)	Kawal	Herb	Seed	Jaundice	Powder mixed with fresh milk, potions	0.39
32	*Tephrosia purpurea* (L.) Pers. (HB/TP0522)	Mardoyaa	Herb	Whole Plant	Wounds	Poultice	0.36
33	*Trigonella foenum-graecum* L. (HB/FF1121)	Hilaba	Herb	Seed	Stomach pain	Infusion, potions	0.58
					Diabetes	Raw, swallow	
34	*Vachellia nilotica* subsp*. adstringens* (Schumach.) Kyal. & Boatwr. (HB/VN1121)	Sunut	Tree	Leaf Fruit	Abscess Corona virus	Poultice Decoction, potions	0.89
35	*Vachellia oerfota* var. *oerfota* (HB/VO1121)	El Ifein	Shrub	Fruit Root	Tooth ache Snake bite Scorpion sting	Poultice Freshly crush, rub Poultice	0.81
	**Lamiaceae**						
36	*Ocimum basilicum* L. (HB/OB1221)	Fillyia	Herb	Whole plant	Rheumatic pain Evil eye	Smoke fumigant	0.49
	**Lythraceae**						
37	*Punica granatum* L	Roman	Shrub	Fruit	Giardia	Maceration, potions	0.35
	**Malvaceae**						
38	*Adansonia digitata* L. (HB/AD0622)	Tabaldi/go ngolase	Tree	Fruit pulp	Diarrhoea	Maceration, potions	0.73
39	*Corchorus trilocularis* L. (HB/CT0122)	Khudra	Herb	Seed	Tonsillitis	Decoction, rinse	0.17
40	*Grewia tenax* (Forssk.) Fiori (HB/GT0622)	Guddaim	Shrub	Fruit	Anaemia	Maceration, potions	0.74
				Root	Abscess	Poultice	
41	*Hibiscus sabdariffa* L. (HB/HS0422)	Karkade	Herb	Cayx	Hypertension	Maceration, potions	0.92
					Cough/ Flu	Maceration, potions	
	**Meliaceae**						
42	*Azadirachta indica* A.Juss. (HB/AI1121)	Neem	Tree	Leaf	Rheumatic pain	Maceration, wash	0.49
	**Molluginaceae**						
43	*Mollugo cerviana* (L.) Ser. (HB/MC1121)	Al Kashibbra	Herb	Whole plant	Kalaazar Lip dermatitis	Poultice Decoction, rinse	1.01
					Wound	Poultice	
	**Moraceae**						
44	*Ficus sycomorus* L. (HB/FS0422)	Guomaze	Tree	Bark	Gum inflammation	Decoction, wash	0.29
	**Myrtaceae**						
45	*Eucalyptus globules* Labill. (HB/EG1121)	Ban	Tree	Leaf	Hypertension	Decoction, potions	0.48
46	*Syzygium aromaticum* (L.) Merr. & L.M.Perry	Gronful	Tree	Flower bud	Corona virus	Decoction, potions	0.24
	**Olacaceae**						
47	*Ximenia americana* L. (HB/XA1121)	Beu	Tree	Root bark	Rheumatic pain	Powder mixed with seasam oil and rubbed	0.43
				leaf	Measles	Decoction, wash	
	**Orobanchaceae**						
48	*Striga hermonthica* (Delile) Benth. (HB/SH0122)	Buda	Herb	Whole plant	Urine retention	Ash, decoction, potions	0.65
					Menstrual cramps	Decoction, potions	
	**Pedaliaceae**						
49	*Sesamum indicum* L. (HB/SI0722)	Simsim	Herb	Seed	Head ache	Oil, rub	0.48
	**Poaceae**						
50	*Chrysopogon nigritanus* (Benth.) Veldkampis (HB/CN0122)	Irg almouya	Herb	Root	Diarrhoea	Infusion, potions	0.16
51	*Cymbopogon schoenanthus* Spreng. (HB/CS0122)	Marhabab	Herb	Whole Plant	Abdominal pain	decoction, Potions	1.01
					Women infertility	Decoction, potions	
					Renal colic	Decoction, potions	
52	*Eragrostis cilianensis* (All.) Vignolo ex Janch. (HB/EC0722)	Banoo	Herb	Root	Dyspepsia	Raw (chewed fresh)	0.10
	**Ranunculaceae**						
53	*Nigella sativa* L	Al haba elsowda	Herb	Seed	Corona virus	Decoction, potions	0.92
					Diabetes	Decoction, potions	
					Head ache	Powder, inhalation	
					Prostate	Decoction, potions	
	**Rhamnaceae**						
54	*Ziziphus spina-christi* (L.) Willd. (HB/ZS1121)	Nabk Karno	Tree	Bark	Kidney stones	Maceration, potions	0.81
				Leaf	Evil eye	Smoke fumigant	
	**Rubiaceae**						
55	*Vangueria madagascariensis* J.F.Gmel. (HB/VM1221)	Kir kir	Tree	Fruit	Hypertension	Maceration, potions	0.24
	**Solanaceae**						
56	*Solanum coagulans* Forssk. (HB/SC0422)	Gabean	Herb	Seed	Abdominal pain	Raw, swallow	0.35
57	*Solanum forskalii* Dunal (HB/SF0422)	Aldayok	Shrub	Fruit	Head pustules	Poultice	0.81
					Head ache	Infusion, potions	
				Seed	Snake bite Malaria Luck	Freshly crush, rub Infusion, potions Powder	
	**Zygophyllaceae**						
58	*Balanites aegyptiaca* (L.) Delile (HB/BA1121)	Hagleeg	Tree	Bark	Rheumatic pain	Smoke fumigant	1.57
				Fruit pulp	Jaundice	Maceration, potions	
					Diarrhoea	Maceration, potions	
					Dysentery	Maceration, potions	
					Stomachache	Maceration, potions	
59	*Tribulus terrestris* L. (HB/TT0722)	Dreesa	Herb	Fruit	Kidney stones	Maceration, potions	0.58

Forty-five medicinal uses were recorded. The most frequently claimed medicinal uses were for the digestive system (16 plants, 7 uses), skin diseases (14 plants, 6 uses), while urinary (4 uses), respiratory systems (6 uses) and parasite infections (6 uses) were treated by 9 plants each. Poisonous animal bites (3 uses) were treated by 7 plants. Gynaecological diseases (6 uses) and muscolo-skeletical (1 use) diseases were treated by 6 plants each. Five plants each to treat blood system disorders (2 uses), pain (headache and teeth ache) and diabetes, while only one plant was reported for swellings.

### Habitat of the plants

In terms of life form, analysis of data showed that herbs accounted for the highest proportion (30, 50.8%) followed by trees (21, 35.6%) and shrubs (8, 13.6%), respectively.

### Parts of medicinal plants used

Analysis showed that informants use various parts of medicinal plants. Stem bark and fruits contributes about (12, 17.9% each), followed by whole plant and roots (11, 16.4% each), seeds (9, 13.4%), leaves (8, 11.9%), flowers (bud/calyx) and gum (2, 3% each), respectively.

### Mode of preparation and path of administration

**Fig. 2:**
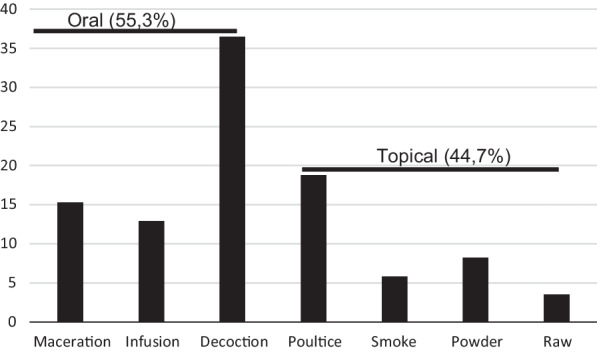
Mode of preparation of herbal drugs

The informants prepared their remedies in various forms including decoction (31, 36.5%), poultice (16, 18.8%), maceration (13, 15.3%), infusion (11, 12.9%), or applied as smoke (5, 5.8%) powder (6, 8.2%) or taken as raw (3, 3.5%) from dried and subsequently collected plant parts (Fig. 2). 55.3% of preparations were orally administrated, while 44.7% were externally applied.

### Quantitative analyses of ethnomedicinal data

#### Most frequently cited plant species

Score of use value (UV) ranged between 0.10–2.37 with the highest value recorded for *Geigeria alata* (Table 1). *Balanites aegyptiaca* (UV = 1.57), *Senna alexandrina* (UV = 1.17), *Boswellia papyrifera* (UV = 1.15), *Mollugo cerviana* (UV = 1.01) and *Blepharis eilensis* (UV = 1.00) have also high UV indicating their lead position in terms of popularity and significance application in local practice. In contrast, *Eragrostis cilianensis* (UV = 0.10), *Vangueria madagascariensis* and *Syzygium aromaticum* (UV = 0.24) recorded the lowest UV values suggested their lower medicinal value appreciation.

#### Informant consensus factor (ICF) and fidelity level (FL)

Plants were assembled into 12 categories and the ICF was calculated and is presented in Table 3. ICF values ranged between 0.43 and 1.00. The highest ICF values are recorded for swellings (ICF = 1.00) and respiratory system (ICF = 0.95) categories. The category of plants used for treatment of skin diseases has the lowest degree of consensus (ICF = 0.43). The FL values were calculated for the most important plant in each ailment category (Table 3). FL values were in the range of 73.33–100.00. Highest FL was recorded for *Blepharis linariifolia, Geigeria alata. Senna alexandrina* and *Psiadia punctulate* (FL = 100)*.*

**Table 3:** Diseases categories and preferred species application by informant consensus factor (ICF) and fidelity level (FL)

Ailments category	*N*_t_	*N*_ur_	ICF	Preferred species	Application	Fl (%)
Respiratory system	9	120	0.95	*Vachellia nilotica*	Corona virus	90.47
Cardiovascular system	5	27	0.85	*Hibiscus sabdariffa*	Hypertension	89.47
Digestive system disorders	16	42	0.63	*Senna alexandrina*	Constipation	100
Urinary system	9	33	0.75	*Blepharis linariifolia*	Urine retention	100
Genital system	6	22	0.76	*Striga hermonthica*	Menstrual cramps	73.33
Infections/infestations	7	16	0.60	*Balanites aegyptiaca*	Dysentery	90.47
Skin diseases	14	24	0.43	*Mollugo cerviana*	Wounds	93.75
Muscle-skeletal system	6	24	0.87	*Azadirachta indica*	Rheumatic pain	73.33
Endocrinology system (diabetes)	5	31	0.87	*Geigeria alata*	Diabetes	100
Abnormalities	1	29	1.00	*Psiadia punctulate*	Swellings	100
Pain (tooth pain, head ache)	5	15	0.71	*Nigella sativa*	Head ache	80.00
Bites	7	27	0.77	*Vachellia oerfota*	Snake bite	75.00

Respiratory system diseases: cough/cold/flu, corona virus, catarrh and tonsillitis. Cardiovascular system and hematological disorders: anaemia and hypertension. Digestive system disorders: stomachache, abdominal pain, diarrhoea, constipation, haemorrhoids, dyspepsia, and jaundice. Urinary system disorders: renal colic, urine retention and kidney disorders. Genital system: women infertility, menstrual cramps, abortion, prostate, syphilis, and gonorrhoea Infections/infestations: malaria, fever, bilharzia, dysentery, giardia, worm expulsion kalaazar and gum inflammation. Skin diseases: wounds, measles, head pustules and eczema. Musculoskeletal system: rheumatism. Endocrinological system: diabetes. Abnormalities: swellings. Pain: headache and toothache. Bites: scorpion sting, snake bites and rabies

*N*_*t*_ number of taxa, *N*_*ur*_ number of use-reports

## Discussion

### Demographic features of informants and source of knowledge

A total of 135 informants were interviewed regarding the use of medicinal plants to treat different ailments. Women play significant role in the traditional medicinal system of Melit area, and the slightly higher number of male informants (55.56%) was attributed to the fact that men can travel long distances for the collection of the medicinal plants. Diagnostic assessment involves patient self-reporting, observation, questioning, listening, smelling and palpating. There are no fixed fees for curing with traditional system, patients pay a symbolic price and, in most cases, it is free of charge. In fact, the revenue generated from the practice of traditional medicine is not the primary source of income to healers. The majority of informants were illiterate, and data on age group indicated that old people have much experience on curing diseases by medicinal plants. Also, the number of informants decreased among educated ones, suggesting that education seems to have an inverse effect on practicing traditional medicine. Oral transmission from one generation to other (89.63%) was the main way through which informants acquired their knowledge on curing diseases by medicinal plants. These demographic characteristics of informants were also in agreement with previous reports in other regions of Sudan [17–23] and the world like Ethiopia [1], India [2] and Pakistan [24] among others. Additionally, traditional healers in general are complacent with their practice among their communities without formal or legal recognitions. Thus, the government, scholars, communities and knowledge bearers should work together to protect the endangered traditional medicine culture through documentation, provide training and education to younger generations and creating strategies for the preservation of such important cultural heritage.

### Ethnobotanical diversity

Ethnobotanical survey in the local community of Melit in north Darfur State (Western Sudan) reported about 59 medicinal plants with 45 medicinal uses recorded from 135 informants. The plants belong to 32 families, and the family Fabaceae is represented by the highest number of species (13) in accordance with previous ethnobotanical studies in other regions of Sudan [17–23]. Herbaceous were the most used plants, and this could be due to their high abundance and easy collection. In fact, it was reported that herbaceous species accounted for 60% of native flora of Sudan, while woody species forming about 30% [5]. Herbs are mainly used in dried form as they are seasonally distributed. The majority of preparations were based from a single plant to cure many diseases suggesting the presences of several bioactive agents that can be effective against several disorder conditions. For example, *Citrullus colocynthis* is used to treat rheumatic pain, malaria, gonorrhoea and scorpion sting. *Nigella sativa* for corona virus, diabetes, head ache and prostate and *Solanum forskalii* for head diseases, malaria and snake bite. Also, in some cases a mixture of more than one plant is used to treat specific disease like malaria is treated with a combination of seeds of *Solanum forskalii* and *Citrullus colocynthis*. Healers in different African countries believe that the body requires treatment with several different plants to produces a healing effect either through complementary benefits or through synergistic effect [19]. In addition, magic and spirituality are well rooted in the Sudanese society and are often used in a mixed way in traditional medicine. The society here believes in evil eye, curse, satan strike and devil interference in people’s lives. Mental disorders and psychological problems are often attributed to act of genie or a curse. One of the methods they use for expelling evil spirit and genie is a blend of gum and dried plants burned in an incense burner made of clay from which smoke will rise and the patient is then exposed to that smoke. For bringing luck or attracting love, healers consider the skin route and therefore prescribe other special herbs worn around the arm. In this study, three plants, namely; *Cassia arereh*, *Ocimum basilicum,* and *Ziziphus spina-christi*, are used for the treatment of evil eye, while *Solanum forskalii* is used to bring chance. Stem bark and fruits followed by whole plant and roots were the most used parts for herbal preparations in agreement with ethnobotanical studies from Kordofan States (Western Sudan) [21, 23], Blue Nile State (South Eastern Sudan) [19] and contrary to results from other regions in Sudan where leaves were usually the favoured part [20, 22]. In most instances the same plant’s part being used for different purposes. Decoction is the most mode of preparation used and informants believed that heat better release bioactive components of the plants in water and also to avoid microbial attack. Healers are also aware of the correlation of the dose given to the age, physical and health conditions of patients. Also, some rituals that believed to have beneficial effect are also performed. Oral preparation is the main administration rout in most herbal remedies and additives like milk or honey or oil are frequently used to improve the acceptability of certain oral remedies in line with previous reports [17–23].

### Comparative review of traditional usages of reported species with previous studies from Sudan

Comparison with all previous ethnobotany studies carried in Sudan as well as those reported in the Atlas for Medicinal Plants of the Sudan was done and summarized [17–23, 25–29] (Table 4). It was noted that many plant species identified in the present study were also reported with the same uses in other regions of the Sudan suggesting their reliable curative effects and also reflecting high cultural exchange between local communities in different regions of the Sudan [30]. Also, there are some species with different uses, like for example *Leptadenia arborea* is used to treat kidney stones in the present study, while in other regions of Sudan it is used against acid reflux, diarrhoea, swellings, dandrof and jaundice [18, 19, 21, 23, 27, 28]. Also, *Aloe sinkatana* is used to cure tonsillitis, while in other regions of Sudan it is used to treat wounds and headache [22, 25]. *Eucalyptus globules, Hyphaene thebaica* and *Vangueria madagascariensis* are used to treat hypertension in the present study, while in other regions of Sudan they are used to treat other diseases like diabetes, diarrhoea, kidney stones and wound [19, 21, 23, 25–29]. Ten plants, namely *Carica papaya, Corchorus trilocularis, Eragrostis cilianensis, Heliotropium sudanicum, Mollugo cerviana, Psiadia punctulate, Rhynchosia minima, Solanum coagulans, Solanum forskalii* and *Tephrosia purpurea,* were cited for the first time as medicinal plants used in Sudan traditional medicine. However, their ethnobotanical uses in other cultures around the world as well as their studied biological activities and phytoconstituents are summarized in Table 5. It was noted that no ethnobotanical uses and scientific studies were reported for *Eragrostis cilianensis, Heliotropium sudanicum* and *Solanum forskalii*, and thus, they are worth further studying over their phytochemical and biological properties. For other species, most of them have different traditional uses from those reported in the present study except for *Carica papaya* and *Tephrosia purpurea.* The former is also used in many countries like Nigeria, Philippines and India to treat rheumatism and skin disorders (Table 5). *Tephrosia purpurea,* which is also used in India to heal wounds, was found to possess wound healing potential by enhancing the fibroblast cells, collagen fibres and blood vessels formation [31]. Furthermore, a study on *Psiadia punctulate,* which is used to treat swellings in the current study, showed that the sesquiterpene 1β-hydroxy-8-oxo-cyperone (isolated from this plant) has significant antiproliferative activity towards Jurkat and HeLa (IC_50_ = 12 and 18 µM, respectively) cells [32]. *Mollugo cerviana*, which is used to treat some skin disorders, was shown to possess potent anti-inflammatory property in the in vitro acute inflammation model of LPS-stimulated RAW 264.7 cells [33].

**Table 4:** Comparative review of traditional usages of reported species with previous studies from Sudan

Plant species	Diseases treated in current study	El Ghazali et al. [25–29]	EL-Kamali [17]	Koda and Yagi [18]	Musa et al. [19]	Suleiman [20]	Issa et al. [21]	Adam et al. [22]	Eisawi et al. [23]
*Adansonia digitata*	Diarrhoea	Stomachache []^26^	Fever Diarrhoea	Pain after birth	Malaria Diarrhoea Dysentery	Dysentery Diarrhoea Stomachache Fever Kidney stones	Giardiasis Stomachache	–	Giardiasis Stomachache
*Albizia anthelmintica*	Anthelmintic	Stomachache [26]	Anthelmintic	Anthelmintic	–	Anthelmintic	Anthelmintic Wounds Stomachache Jaundice	–	Anthelmintic Wounds Stomachache Jaundice
*Allium sativum*	Haemorrhoids	Haemorrhoids [27]	–	–	–	–	Haemorrhoids	–	Haemorrhoids
*Aloe sinkatana*	Tonsillitis	Wounds [25]	–	–	–	–	–	Wounds Headache	–
*Ambrosia maritima*	Diarrhoea	Swellings [25, 26]	–	–	–	–	–	–	–
*Aristolochia bracteolata*	Malaria Tooth ache Scorpion sting	Malaria [25] Swellings [27] Scorpion sting [28]	Scorpion sting	–	Malaria	Malaria HIV-1 Scorpion sting Ear infection Wounds	Malaria Ear infection Headache	–	Malaria Ear infection Headache
*Azadirachta indica*	Rheumatic pain	Fever [26] Scorpion sting [26] Snake bite [27] Intestinal spasm [26] Anthelmintic [29] Constipation [28]	Antipyretic Backach	–	Malaria Fever Jaundice	–	Rheumatic pain Malaria	–	Rheumatic pain Malaria
*Balanites aegyptiaca*	Rheumatic pain Jaundice Diarrhoea Dysentery Stomachache	Constipation [26, 27] Bilharzia [27] Wounds [27]	Diabetes	–	–	Stomachache Anthelmintic Dysentery Constipation Jaundice Diabetes	Hypertension Bilharzia Jaundice	–	Diabetes Hypertension Bilharzia Jaundice
*Bauhinia rufescens*	Diabetes	Tooth paste [26] Diabetes [25, 27]	–	Cough	Dysentery	–	–	–	–
*Blepharis eilensis*	Urine retention	Stomach pain [26] Bilharzia [26]	Kidney stone Stomach pain	Urine retention	–	Swellings	Kidney disorders Diabetes Wounds Hypertension Toothache Tonic	–	Kidney disorders Diabetes Wounds Hypertension Toothache Tonic
*Boswellia papyrifera*	Corona virus Cold/cough Diabetes	Jaundice [26]	–	Dysentery Respiratory infections	Bilharzia Diarrhoea Dysentery	–	Diabetes Diarrhoea	–	Diabetes Diarrhoea Anaemia
*Cadaba glandulosa*	Kalaazar Wound	Rheumatic [29] Pain [25] Swellings [25, 27]	–	–	–	–	–	–	–
*Calotropis procera*	Wounds Haemorrhoids	Wounds [26] Rheumatic pain [26] Scorpion sting [28] Jaundice [28]	Haemorrhoids Scorpion sting	Scorpion sting Rheumatic pain	–	Scorpion sting Haemorrhoids Rheumatic pain Wounds	Scorpion sting Wounds	–	Scorpion sting Wounds
*Carica papaya*	Eczema	–	–	–	–	–	–	–	–
*Cassia arereh*	Malaria Evil eye	–	–	–	Stomachache Diarrhoea Evil eye	–	Stomachache Malaria Toothache Haematuria Evil eye	–	Stomachache Malaria Toothache Haematuria Evil eye
*Chrysopogon nigritanus*	Diarrhoea	Diarrhoea [27]	–	–	–	–	–	–	–
*Citrullus colocynthis*	Rheumatic pain Malaria Gonorrhoea Scorpion sting	Swellings [27] Purgative [25, 28] Gonorrhoea [28] Diabetes [27] Snake bite [26] Scorpion sting [26]	–	–	–	–	–	Skin blemishes Skin allergies	–
*Corchorus trilocularis*	Tonsillitis	–	–	–	–	–	–	–	–
*Cordia sinensis*	Wounds	Cuts [26] Burns [26] Wounds [26]	Cuts, Burns Wounds	–	–	Cuts, Burns Wounds	–	–	–
*Cymbopogon schoenanthus*	Abdominal pain Women infertility Renal colic	Stomachache [26]	–	–	–	Antispasmodic Stomachache Gout Helminthiasis Inflammation of prostate	Diabetes	–	Diabetes Stomachache
*Eragrostis cilianensis*	Dyspepsia	–	–	–	–	–	–	–	–
*Eucalyptus globules*	Hypertension	Diabetes [27]	–	–	–	–	–	–	–
*Ficus sycomorus*	Gum inflammation	–	–	–	Stomach ache Skin rashes	–	–	–	–
*Foeniculum vulgare*	Stomach ache	Stomach ache [27] Acid reflux [27] Flatulence [27]	–	–	–	–	–	–	–
*Geigeria alata*	Diabetes Catarrh	Stomach ache [26] Epilepsy [27]	Diabetes Antispasmodic Intestinal complaints Hypertension Cough	–	–	Antispasmodic Stomach ache Intestinal complaints Anthelmintic Diabetes Hypertension Cough	Diabetes Stomach ache Kidney disorders Hypertension	–	Diabetes Stomach ache Kidney disorders Hypertension
*Grewia tenax*	Anaemia Abscess	Tonsillitis [26] Swellings [26] Jaundice [27] Trichoma [27]	–	–	–	Tonsillitis Throat Infections Anaemia Malaria Tonic	Wounds Anaemia	–	Wounds Anaemia
*Heliotropium sudanicum*	Scorpion bite	–	–	–	–	–	–	–	–
*Hibiscus sabdariffa*	Hypertension Cough/ Flu	–	Snake bite Scorpion sting Haemorrhoids Headache	–	–	Cough Headache Haematuria Fever Hypertension Snake bite Scorpion sting	Hypertension Cough/ Flu Haemorrhoids	–	Hypertension Cough/ Flu
*Hyphaene thebaica*	Hypertension	Spleen [27] Problems [28] Stomach ache [28] Wound [28]	–	–	–	–	Diabetes Diarrhoea Kidney stones	–	Dysentery Diabetes Diarrhoea Kidney disorders
*Leptadenia arborea*	Kidney Stones	Snake bite [27] Gonorrhoea [28] Swellings [28]	–	Jaundice Dandruff	–	Jaundice Dandruff	Acid reflux Diarrhoea Swellings Jaundice	–	Acid reflux Diarrhoea Swellings Jaundice
*Maerua crassifolia*	Wound	–	–	Wound	–	–	–	–	–
*Mollugo cerviana*	Kalaazar Lip dermatitis Wound	–	–	–	–	–	–	–	–
*Momordica dioica*	Abortive	Abortive [25]	–	–	-	-	-	-	-
*Nigella sativa*	Corona virus Diabetes Head ache Prostate	Diabetes [28] Hypertension [28] Stomachache [28]	–	–	–	–	Articulation pain Stomachache Headache Jaundice	–	Articulation pain Stomachache Headache Jaundice
*Ocimum basilicum*	Rheumatic pain	–	–	–	–	Eye infection	–	Jaundice Kidney disorders	–
*Psiadia punctulata*	Swelling	–	–	–	–	–	–	–	–
*Pulicaria crispa*	Stomach pain	–	–	–	–	–	–	–	–
*Punica granatum*	Giardia	–	–	–	–	–	–	Diarrhoea Dysentery	–
*Raphanus sativus*	Kidney Stones	–	–	–	–	–	–	–	–
*Rhynchosia minima*	Snake bite Rabies	Anti-acid [25]	–	–	–	–	Snake bite	–	Snake bite
*Senegalia mellifera*	Syphilis	–	–	–	–	Pneumonia Stomachache Heartburn Syphilis Malaria	–	–	–
*Senegalia senegal*	Kidney disorder	–	–	Giardiasis	Kidney problems	Rheumatoid arthritis	Haematuria	–	–
*Senna alexandrina*	Constipation	Carminative [25] Stomachache [27] Laxative [27]	–	–	–	Stomachache Laxative	–	Diabetes	–
*Senna italica*	Constipation	Rheumatic pain [27]	–	Constipation	–	–	Dysentery Laxative Eczema	–	Dysentery Laxative Eczema
*Senna obtusifolia*	Jaundice	Jaundice [27] Wounds [25]	Backache Hypertension	Diabetes Gonorrhoea Intestinal ulcer	Jaundice	Jaundice Laxative	Jaundice Laxative	–	–
*Sesamum indicum*	Head ache	Swellings [27]	–	–	–	–	–	–	–
*Solanum coagulans*	Abdominal pain	–	–	–	–	–	–	–	–
*Solanum forskalii*	Head pustules Head ache	–	–	–	–	–	–	–	–
*Striga hermonthica*	Urine retention Menstrual cramps	Leukoderma [27]	Diabetes	–	–	–	Menstrual Cramps Diabetes	–	Menstrual cramps
*Tephrosia purpurea*	Wound	–	–	–	–	–	–	–	–
*Terminalia brownii*	Rheumatic pain	Diabetes [25] Cough [26]	–	–	–	–	Jaundice Rheumatic pain Wound	–	–
*Tribulus terrestris*	Kidney stones	–	–	–	–	–	Kidney disorders Diabetes	–	Kidney disorders Diabetes
*Trigonella foenum*	Stomach pain Diabetes	Swellings [28] Haemorrhoids [29]	–	–	–	–	Uterus Inflammation Swellings Foot pain	–	Uterus inflammatio
*Vachellia nilotica*	Abscess Corona virus	Cold and flu [27, 28] Tonsillitis [26]	Hypertension	Cough	Phlegmatic Cough Furuncles Malaria	Cold and flu Pharyngitis	Stomachache	–	–
*Vachellia oerfota*	Tooth ache Snake bite Scorpion sting	Swellings [28] Scorpion sting [28]	–	Tooth cavity	Toothache Headache Snake bite	Antirheumatic	Back pain Swellings Snake bite Toothache	–	–
*Vangueria madagascariensis*	Hypertension	–	–	–	Diabetes	–	Diabetes	–	Diabetes Kidney disorders Hypertension
*Ximenia americana*	Rheumatic pain Measles	Measles [25]	–	Rheumatic pain	–	–	Rheumatic pain	–	Rheumatic pain
*Ziziphus spina*–*christi*	Kidney stones Evil eye	Swellings [26] Constipation [26] Intestinal spasm [27] Stomachache [28] Gonorrhoea [28]	Antispasmodic Fever	–	Stomachache Dysentery Diarrhoea Malaria Urine retention	Swellings Antispasmodic Constipation Gonorrhoea	Stomachache Dysentery Evil eye	–	Stomachache Dysentery Evil eye

**Table 5:** Worldwide traditional usages, biological activity and phytoconstituents of the plants that are reported for the first time in Sudan traditional medicine

No.	Plant name	Country	Part used	Uses	Biological activity	Phytoconstituents	References
2	*Carica papaya*	Gambia Nigeria Malaysia Philippines Japan India Pakistan	Fruit Leaf	Paediatric burns Diabetes Jaundice Rheumatism Malaria Hypertension Ulcer Digestive disorders Urinary tract infection Skin diseases Dengue fever	Antioxidant Anthelmintic Wound healing Antimicrobial Antidiabetes	Flavonoids Phenolic Acids Glucosinolates Cyanogenic glucosides Alkaloids Saponins Triterpenoids	[38–43]
2	*Corchorus trilocularis*	Pakistan India	Seed Leaf Root	Syphilis Demulcent, Fever Haemorrhoids Laxative	Antiinflammatory Antioxidant Antihyperglycaemic Antipyretic analgesic	Flavonoids Triterpenes	[44, 45]
3	*Eragrostis cilianensis*	**–**		No report	No report	No report	
4	*Heliotropium sudanicum*	**–**		No report	No report	No report	
5	*Mollugo cerviana*	India		Rheumatism Haemorrhoids Fever Skin diseases Snake bite Jaundice	Antioxidant Antimicrobial Antiinflammatory	Phenols Flavonoids Terpenoids Steroids Alkaloids Saponins	[33, 46]
6	*Psiadia punctulate*	Kenya	Leaf Root	Colds Fevers Asthma Malaria Abdominal pains Skin Infection	Antimicrobial Antiplasmodial Antitrypanosomal Antiproliferative	Flavonoids Terpenoids Coumarins	[32]
7	*Rhynchosia minima*	Zimbabwe China South Africa	Root Leaf	Skin diseases Respiratory Swelling Joint pains Heart or chest pain	Antimicrobial	Flavonoids	[47]
8	*Solanum coagulans*	China	Aerial parts	Oedema Rheumatic arthritis Toothache	Antifungal Antibacterial	Phenolic glycoside	[48]
9	*Solanum forskalii*	**–**		No report	No report	No report	
10	*Tephrosia purpurea*	India Sri Lanka Ceylon	Root Leaf	Wounds Gastro-duodena disorders Dyspepsia Diarrhoea Haemmaroids Asthma Anaemia Fever Syphills Gonnorhea Snake bites Nematicide Anthelmintic	Antiulcer Antitumor Antimicrobial Antiinflammotory Antioxidant Hepatoprotective Antihyperlipidimic Antihyperglycemic Anthelminitic Antileishminal Antidiarrheal Wound healing Spasmolytic	Flavonoids Sterols Terpenes	[31, 49]

### Frequent diseases and cited medicinal plants

Medicinal uses are distributed into 12 categories of ailments, and analysis revealed that the digestive system (16 plants, 7 uses), skin diseases (14 plants, 7 uses), urinary and respiratory systems (9 plants each, 5 uses), respectively, were the most frequently claimed medicinal uses, suggesting that these diseases were more likely the prevalent disease in the area. Moreover, the majority of ailment categories has ICF ≥ 0.71 indicating high degree of consensus between informants [34]. UV ranged between 0.10 and 2.37 (Table 1). Medicinal plants with high UV have usually more use-reports and high availability and importance [35]. On the other hand, attention should be considered for plants with low UV as their less use might increase the risk of disappearing of their curative knowledge. *Blepharis linariifolia, Geigeria alata. Senna alexandrina, Psiadia punctulate, Mollugo cerviana, Balanites aegyptiaca* and *Vachellia nilotica* were the most preferred species as they have high FL. In fact, these species except *Mollugo cerviana* and *Psiadia punctulate* are reported to have the same traditional uses in other regions of the Sudan [30]. Additionally, there are many scientific evidences supporting their traditional uses. For example, *Geigeria alata* that is used to treat diabetes has been proven to significantly reduced the serum glucose level in diabetic rats and to possess α-glucosidase inhibitory and pancreatic lipase inhibitory activities [36]. *Balanites aegyptiaca* that is used to treat rheumatic pain, jaundice, diarrhoea and dysentery is found to exert antioxidant, anti-inflammatory, anticancer, antinociceptive, hepatoprotective, hypocholesterolemic, diuretic, antibacterial, antiviral and anthelmintic activities [37]. *Senna alexandrina* is well known for its laxative effect since ancient time. Moreover, during the pandemic of COVID-19 which caused the death of hundreds of people in Sudan, informants used a number of plants. For example, they used *Boswellia papyrifera* resins, seeds of *Nigella sativa* and pods *Vachellia nilotica* (syn. *Acacia nilotica*) to make different preparations for the treatment of the virus. Another recipe is a mixture of clove (*Syzygium aromticum*) decoction and honey with lemon and lemon peel.

### Endangered medicinal plants

The majority of interviewee declared that, generally the availability of medicinal plants is declining. They reported *Blepharis linariifolia, Cadaba glandulosa, Cordia sinensis* and *Adansonia digitate* as the most endangered plants. This was attributed to overgrazing, fires, exploitation of forest for biomass for energy in addition to general environmental degradation. It is noteworthy that drought is a major problem experienced by Sudan and has resulted in an alarming depletion of the biodiversity. The natural and human-induced rapid environmental change decreased the availability for certain medicinal plants from the wild, besides, there is no cultivation practice to these medicinal plants. All these factors may represent a serious challenge to the continuity and efficacy of traditional medicine in the study area.

## Conclusion

The present ethnobotanical survey indicated that knowledge of traditional medicine is highly valued in the community of Melit and it symbolizes culture identity and a source of community pride. A considerable number of plants have emerged from this survey reflects evidence that Melit area harbours a high diversity of medicinal plants that will continue to play an important role in the healthcare system in the area. The majority of medicinal plants were mainly distributed in the wild, with the fruits and stem bark being the most used parts and the primary preparation method being decoction. Results also revealed that 45 diseases were treated with medicinal plants, with ailments related to the digestive system being the most common. The present study aids in conserving such rich heritage and providing precious information as a contribution through writing the Sudanese pharmacopoeia. Anthropogenic disturbances and environmental factors are the major threat and challenge facing medicinal plants and traditional healing culture in the Sudan. Memorization may not be sufficient to preserve traditional knowledge on medicinal plants, besides, the disappearance of some plants may become a threat for the traditional knowledge on medicinal plants. Therefore, it is very crucial that awareness creation to be undertaken so that the community is actively involved in conservation of this knowledge and sustainable utilization of the traditional medicinal plants. Furthermore, an important concern in the therapeutic use of some plants is their toxic side effect. Among the plants established to be toxic and cancerogenic is *Aristolochia bracteolate*, due to its content of aristolochic acids, which called for strict control on the use of the plant. Also, there is high need for scientific research and development with a view to set standard products in the international market parallel with plans for large scale systematic processing and value-added up-scaling.

## Data Availability

We have already included all data in the manuscript collected during the field surveys.

## Abbreviations

ICF: Informant consensus factor
UV: Use value
FL: Fidelity level
